# Management of Monochorionic Twin Pregnancies Complicated by Selective Fetal Growth Restriction: Retrospective Single-Center 12-Year Experience

**DOI:** 10.3390/diagnostics15202653

**Published:** 2025-10-21

**Authors:** Sofia Roero, Silvana Arduino, Agata Ingala, Carlotta Bossotti, Isabella Ferrando, Miriam Folino Gallo, Chiara Peila, Alessandra Coscia, Alberto Revelli

**Affiliations:** 1 Twin Pregnancy Care Unit, Gynecology and Obstetrics 2U, A.O.U. Città della Salute e della Scienza, Sant’Anna Obstetric Gynecological Hospital, University of Turin, Corso Spezia 60, 10126 Turin, Italy; 2 Department of Public Health and Pediatrics, A.O.U. Città della Salute e della Scienza, University of Turin, Via Santena 5, 10126 Turin, Italy

**Keywords:** twin pregnancy, growth restriction, ultrasound

## Abstract

**Objectives:** To describe the perinatal outcomes of a series of monochorionic diamniotic (MCDA) twin pregnancies complicated by selective fetal growth restriction (sFGR), classified according to the umbilical artery (UA) Doppler flow pattern of the smaller twin, and managed in a single centre over a 12-year period. **Methods:** Retrospective cohort study involving MCDA twin pregnancies followed up at the Twin Pregnancy Care Unit of Sant’Anna Hospital, Turin, Italy, between January 2010 and May 2023. We compared perinatal outcomes of MCDA pregnancies classified based on the UA Doppler flow pattern of the smaller twin. **Results:** The percentage of sFGR in our sample was 14.8%. A total of 103 MCDA pregnancies with sFGR were included. In 34.9% cases, the UA flow pattern changed throughout pregnancy. At last examination, 58.3% cases were classified as type I, 25.2% as type II and 16.5% as type III. The perinatal survival rate of both twins in type I and III was 100%, in type II 88.5%. Type II sFGR had the highest perinatal mortality rate (7.7%). Type III twins were more likely to have malformations compared to type II and type I; compared to type I sFGR babies, they were more likely to develop RDS and to be admitted to NICU, where the length of stay was longer. **Conclusions:** Although the UA flow pattern correlates with perinatal outcome, it can change throughout pregnancy. Type III sFGR may have lower risk of fetal demise than traditionally thought. The main challenge remains finding the optimal balance between adverse outcomes and premature birth.

## 1. Introduction

Selective fetal growth restriction (sFGR) is a complication unique to monochorionic twin pregnancy. According to the Delphi consensus, it is defined as an estimated fetal weight (EFW) of one twin <3rd centile or a combination of two out of the following four parameters: EFW of one twin <10th centile; abdominal circumference (AC) of one twin <10th centile; inter-twin EFW discordance ≥25%; and umbilical artery pulsatility index (UA-PI) of the smaller twin >95th centile [1]. Another definition describes sFGR as a condition in which one fetus has EFW <10th centile and the inter-twin EFW discordance is >25% [2,3].

sFGR affects 10–15% of monochorionic twin pregnancies and significantly increases perinatal mortality and morbidity, especially related to brain damage and neurological impairment [4,5,6,7,8,9,10]. The pathophysiology of sFGR seems tightly associated with placental structure, unequal placental sharing and vascular anastomosis [11]. sFGR is usually classified based on the umbilical artery (UA) flow pattern of the smaller twin [9]: type I is characterized by persistently positive end-diastolic flow and generally has the best prognosis and lowest progression risk. Type II sFGR has a persistently reverse or absent flow pattern and is associated with high risk of fetal demise of the smaller twin. Type III is characterized by an intermittently absent or reversed flow pattern and has an unpredictable prognosis [1,2,9]. This classification, however, does not take into account gestational age at diagnosis or the variation of flow patterns during gestation, both of which may influence perinatal outcomes [12,13,14,15].

The optimal management of sFGR has yet to be determined. Options include expectant management, selective termination by cord occlusion and, in case of superimposed twin-to-twin transfusion syndrome, laser coagulation of placental anastomosis [6,13,16,17,18]. It has been recently reported that selective reduction might be especially beneficial in type II and III sFGR, as it significantly reduces prematurity-related morbidity for the larger twin [19].

The main objective of the present work is to describe perinatal outcomes of a series of monochorionic twin pregnancies complicated by sFGR (classified according to the UA flow pattern of the smaller twin), all followed up in a single centre with the application of a strict, specific prenatal protocol. A secondary objective is to evaluate how often the Doppler flow pattern can change throughout pregnancy and whether it appears to influence the prognosis of these pregnancies.

## 2. Materials and Methods

All women included in this retrospective cohort study carried monochorionic diamniotic (MCDA) twin pregnancies complicated by sFGR. Women were followed up at the Twin Pregnancy Care Unit of Sant’Anna Hospital, Turin, Italy, between January 2010 and May 2023, and were managed by the same team of obstetricians, applying a strict follow-up protocol. Data were gathered through hospital records of these patients. Exclusion criteria comprised the following: monoamniotic twin pregnancy, triplet pregnancy, cases with chromosomal abnormalities and women who delivered in another hospital. Because of anonymous data collection and its retrospective nature, this study was deemed exempt from approval by a local review board. It fully adheres to the World Medical Association Declaration of Helsinki (as revised in 2013) and complies with ethical standards of national and institutional committees on human experimentation. A written informed consent for use of personal information was obtained from every participant using a designated form that was signed at the time of the first visit.

All included women with MCDA twin pregnancy were referred to the Twin Pregnancy Care Unit between 12 and 16 weeks of gestational age. Chorionicity was established in the first trimester applying standard US criteria [2]; chorionicity was confirmed by postpartum placental examination.

Starting from the 16th week onward, MCDA twin pregnancies received serial US assessments including fetal biometry, measurement of deepest amniotic fluid vertical pocket, bladder visualization, placental site assessment and Doppler flow measurement of the UA and middle cerebral artery (MCA) [2,20,21]. UA flow was measured in a free loop, and several waveforms of both UAs were obtained: when flow anomalies were detected, both UAs were sampled at the portion of the umbilical cord closest to placental insertion for confirmation [20]. In the presence of different flow patterns in the two UAs, the worst of the two was used for classification in order not to underdiagnose type II and III sFGR [14]. Doppler evaluation of the ductus venosus (DV) was performed in the smaller twin in the absence of fetal movements, and the a-wave was categorized as positive, negative or reversed. EFW was calculated according to Hadlock [22]; fetal growth charts according to Paladini were used [23].

As recommended by national and regional guidelines [24,25], sFGR was defined as an EFW of one twin <5th centile or a combination of two out of the following four parameters: EFW or AC of one twin <10th centile; intertwin EFW discordance ≥25%; and UA-PI of the smaller twin >95th centile. This represents a slight modification of the Delphi consensus conference definition, which considers EFW <3rd centile [1]; such choice is determined by the fact that our Centre applies national and regional guidelines recommendations, which use the 5th centile as a cutoff. This variation might be associated with a slight overdiagnosis of the cases of sFGR. However, the incidence of this complication in our dataset is not different from that reported in the literature; therefore, it can be concluded that comparability to other international studies is not an issue.

EFW discordance was expressed using the following ratio: ((larger twin EFW − smaller twin EFW)/larger twin EFW) × 100 [26]. After diagnosis, sFGR was classified in type I, II or III according to the Gratacos classification [9] and weekly US examinations were scheduled for measurement of deepest amniotic fluid vertical pocket, bladder visualization and Doppler flow measurement of the UA and middle cerebral artery (MCA); fetal growth was assessed every 2 weeks. Furthermore, an US scan for detailed anatomy, including fetal echocardiogram, was performed at 19–21 weeks to detect anatomical anomalies. All US assessments were performed by the same two expert operators (S.A., C.B.) using a Samsung WS80A (sourced from Samsung Healthcare in Turin, Italy) machine equipped with a 4–8 MHz probe.

Treatment options for sFGR included expectant management with strict US surveillance or active management (selective termination of the sFGR twin or laser-coagulation of placental anastomosis). Expectant management was carried out as an outpatient procedure in most cases. Hospitalization and inpatient intensive monitoring were offered after 32 weeks of GA for type I sFGR and after 28 weeks of GA for type II and III sFGR, with daily cardiotocography and serial Doppler flow assessment every second day. Type I cases were all managed expectantly. In type II and III cases which were managed expectantly, the aim was to go on at least until 32 weeks and, possibly, to reach 34 weeks, whereas in type I cases the aim was to reach 35 weeks of GA. The cutoff of 34 weeks was reached in those cases with no alteration in DV Doppler waveform and no signs of fetal distress at cardiotocographic monitoring. In all cases, the timing of delivery was decided individually based on DV waveform, fetal growth and, for hospitalized patients, cardiotocography [2,18]. Selective termination of the smaller twin was considered before fetal viability (<23 weeks, as per Italian law) in severe cases of type II or III sFGR with anomalies in DV waveform. Laser coagulation of placental anastomosis was offered to cases with superimposed twin-to-twin transfusion syndrome (TTTS). All invasive treatments were performed at Buzzi Hospital (Milan, Italy). Spontaneous miscarriage was defined as death of at least one twin before 22 weeks of GA and fetal demise as death from 22 weeks onward.

As regards neonatal outcomes, respiratory distress syndrome (RDS) was defined as the presence of tachypnea, tachycardia, chest wall retractions, expiratory grunting, nasal flaring and cyanosis for more than two hours after birth, with typical radiological signs and need for supplemental oxygen or ventilatory support. Criteria for sepsis were as follows: clinical signs of sepsis, leukopenia, neutropenia, immature neutrophils, and elevated CRP with (proven sepsis) or without (probable sepsis) positive blood culture. Early onset sepsis was defined by an onset before 72 h of life, whereas late onset sepsis by an onset between 72 h and 28 days of life.

Intraventricular hemorrhage (IVH) was graded according to Papile criteria (IVH with ventricular dilatation—grade III and intraventricular rupture and hemorrhage into the surrounding white matter—grade IV) [27]. Neonatal death was defined as death of at least one newborn up to 28 days postpartum. Perinatal death was defined as the sum of intrauterine and neonatal deaths. Of the 203 liveborn babies included in the study, 12 were lost at follow-up due to lack of data because they were followed up at another centre (6 babies in the type I subgroup and 6 in type II).

Outcome variables of different types of sFGR were compared using the two-tailed Student’s *t*-test for parametric variables and the Mann–Whitney U test for non-parametric variables; categorical data were compared by χ^2^ or Fisher’s exact tests. Odd ratios (ORs) were calculated for categorical data using binomial logistic regression. A *p*-value < 0.05 was considered statistically significant. The SPSS statistical analysis software by SPSS Statistics Inc. (IBM Corp., Armonk, N.Y., USA, Released 2021, IBM SPSS Version 28.0.1.1) was used.

## 3. Results

A total number of 778 MCDA twin pregnancies were followed up at the Twin Pregnancy Care Unit of Sant’Anna Hospital in the period of the study; among these, 115 (14.8%) were complicated by sFGR. After excluding pregnancies for incomplete data availability, a final cohort of 107 MCDA twin pregnancies with sFGR were included in the analysis. One case underwent selective termination by cord occlusion and three cases with superimposed TTTS underwent laser-coagulation of placental anastomosis. The remaining 103 cases were managed expectantly and were included in the analysis.

Table 1 shows maternal characteristics of the population included in the study, divided into subgroups based on the type of sFGR. No significant difference between the three groups was detected.

**Figure 1:**
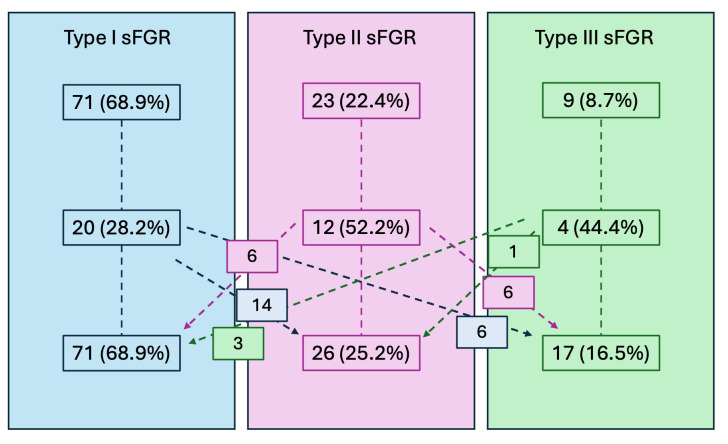
Changes in UA Doppler flow pattern throughout gestation. Data are shown as number and percentage (in parenthesis).

Table 2 shows sFGR classification at the first and last examination in pregnancy. In 36 cases (34.9%), the UA flow pattern changed throughout pregnancy, as shown in Figure 1. The 36 cases that needed reclassification due to a shift in the Doppler flow pattern showed no significant difference compared to those with a stable pattern.

Table 3 and Table 4 show the comparison of obstetric outcomes of type I, II and III sFGR (according to their classification at the last examination). We observed one case of miscarriage (following laser-coagulation of placental anastomosis at 16 weeks) and two cases of fetal demise, all involving the smaller twin in type II sFGR. One case of fetal demise occurred at 25 weeks of gestation, and the co-twin was delivered at 31 weeks because of placental abruption; the other case occurred at 30 weeks and was managed with immediate delivery of the co-twin, also because of suspected placental abruption. The latter newborn developed cerebral venous sinus thrombosis and periventricular leukomalacia because of extra-parenchimal brain hemorrhage, with resulting right hemiparesis.

Among pregnancies complicated by type I sFGR, 46 (76.7%) were delivered at 34 weeks or later.

Table 5 and Table 6 show the comparison of neonatal outcomes of type I, II and III sFGR.

Of the 203 liveborn babies, 12 were lost at follow-up due to lack of data because they were followed up at another centre: data about the remaining 191 newborns are shown. Therefore, around 6% of the babies were lost at follow-up: 6 babies in the type I subgroup and 6 in type II, whilst all babies from type III subgroup are included in the study; such loss is quite small and it is most likely that no selection bias has been introduced in the results because of this. Overall, all 103 women had at least one live birth, with a median gestational age at delivery of 33.5 weeks. Neonatal death occurred in 5/191 twins (2.6%), with an overall survival rate of 98.9% for the larger twin and 95.8% for the smaller one. Of the two cases of neonatal death after type I sFGR, one newborn was born at 30 weeks with severe liver and kidney malformations and died at 20 days of life because of pulmonary hemorrhage; the other case was born at 35 weeks and died because of septic shock. One type II sFGR newborn, born at 28 weeks, died at 19 days because of necrotizing enterocolitis. Of the two type III newborns who died, one, born at 29 weeks, died because of tuberous sclerosis and has been excluded from analysis of perinatal mortality. The other had grade III IVH. Perinatal mortality was 1.7% in type I, 7.7% in type II and 2.9% in type III sFGR.

Our series includes six cases of sFGR with superimposed TTTS, three in the type I subgroup and the three in type II subgroup; no cases were reported in the type III subgroup. Three of the TTTS cases were successfully treated with laser coagulation of placental anastomosis. Another case was diagnosed at 16 weeks and underwent selective termination by cord occlusion. The sixth and fifth case had acute TTTS at 34 and 35 weeks, respectively, and were managed with immediate delivery.

In our series, 14 (13.6%) cases were diagnosed after 24 weeks (late-onset sFGR), whereas the remaining 89 (86.4%) were diagnosed before 24 weeks (early-onset sFGR). No significant differences were found in perinatal outcomes between early- and late-onset sFGR (Table 7). Multivariate logistic regression was performed to explore an influence of GA at diagnosis on pregnancy outcome: no significant association was found.

### 3.1. sFGR Type I vs. Type II

Pregnancies complicated by sFGR type II had higher rates of delivery before 28 weeks (*p* = 0.028; OR = 3.3) and before 32 weeks (*p* < 0.001; OR = 5.6). All twins with sFGR type I survived in utero, compared to 88.5% of twins with type II (*p* = 0.007). Also, perinatal mortality was significantly lower in type I sFGR (1.7% vs. 7.7%, *p* = 0.028; OR = 3.8).

No significant difference was found in fetal weight discordance. As could be expected, sFGR type I larger twins weighed more than type II larger twins (2043 ± 316 g vs. 1595 ± 417 g, *p* < 0.001); the same was found for the smaller twin (1679 ± 313 g vs. 1257 ± 384 g, *p* < 0.001). Neonatal RDS occurred more frequently in type II sFGR babies (*p* = 0.005; OR = 2.9) that were more often admitted to the Neonatal Intensive Care Unit (NICU) (*p* = 0.016; OR = 3.1).

### 3.2. sFGR Type II vs. Type III

No significant difference in obstetric outcomes was found between type II and type III sFGR. All twins with sFGR type III were admitted to NICU, compared to 86% of the twins with type II (*p* = 0.023). Type III twins were also more likely to have malformations compared to type II (*p* = 0007, OR = 5.9), most of which (>85%) involved the heart (septal defects). Perinatal mortality was higher in type II sFGR (7.7% vs. 2.9%, *p* = 0.042, OR = 1.9).

### 3.3. sFGR Type I vs. Type III

Diagnosis of sFGR was made at a significantly earlier gestational age in type III compared to type I (16.6 ± 1.3 weeks vs. 19.7 ± 4.9 weeks, *p* = 0.030). All twins with type I or III sFGR survived in utero. In type III sFGR, gestational age at birth was lower (31.9 ± 1.8 vs. 34.4 ± 1.2, *p* < 0.001), and urgent cesarean section due to CTG anomalies was more frequent compared to type I (41.2% vs. 16.7%, *p* = 0.029; OR = 3.7). Type III sFGR had higher rates of delivery before 32 weeks (82.4% vs. 5.0%, *p* < 0.001; OR = 6.8), although no woman delivered before 28 weeks. Fetal weight discordance was significantly higher in type III twins (24.2% vs. 18.5%, *p* = 0.046). Type I larger twins weighed more than type III larger twins (2043 g ± 316 vs. 1765 g ± 346, *p* = 0.002); the same was observed for the smaller twin (1679 g ± 313 vs. 1236 g ± 299, *p* < 0.001).

Type III sFGR babies had a higher rate of low 1′ and 5′ APGAR score (23.5% vs. 9.6%, *p* = 0.034, OR = 2.8, and 23.5% vs. 8.8%, *p* = 0.021, OR = 3.2, respectively) and were more likely to develop RDS (85.3% vs. 44.7%, *p* < 0.001; OR = 7.2) and to be admitted to NICU (100.0% vs. 66.7%, *p* < 0.001; OR = 2.8), where the length of stay was longer (31.8 vs. 18.7 days, *p* = 0.003). Type III newborns were more likely to develop late onset sepsis (*p* = 0.048; OR = 7.8), IVH (*p* < 0.001; OR = 4.2) and to have malformations (*p* = 0.006; OR = 3.5).

## 4. Discussion

The incidence of sFGR in our dataset was 14.8% of all MCDA twin pregnancies, in the higher end of that reported in the literature [1,2,20,24]. Therefore, despite the slight modification of the Delphi consensus conference definition, it can be assumed that most likely no significant overdiagnosis has been made.

Looking at the last examination in pregnancy, more than half of the cases were classified as type I, one fourth as type II, and one sixth as type III. In our dataset there were fewer type III cases compared to Gratacos [9] and Lopriore [26], who reported an incidence of nearly 40%; our rate was closer to the 14% observed by Ishii [8] and Rustico [14]. We observed a much higher rate of type I cases than all the abovementioned studies (in which it ranged between 30 and 40%). About one fourth of the cases initially classified as type I needed to be reclassified at the end of pregnancy. It is possible that a portion of type-III sFGR cases could be missed in early pregnancy, especially considering longer periods of intermittent pattern, as suggested by Rustico [14]. However, there is also a concrete possibility of a spontaneous shift in the flow pattern throughout gestation, confirmed by the observation of cases initially classified as type III reverting to type I. Overall, our study supports the idea that a shift in UA flow pattern during pregnancy is not unlikely, in agreement with previous studies [14,28,29]. A recent meta-analysis [15] also confirmed this finding and, interestingly, reported that UA Dopplers in type I and III sFGR tend to remain stable, whilst in type II they tend to deteriorate. This possibility should be discussed when counseling patients, and more caution should be exercised in reassuring women with type I sFGR. Indeed, such a shift may have an important impact on perinatal outcome. As previously reported by others, in fact, type I sFGR was confirmed to be the mildest form, with the best perinatal outcomes [1,2,9,14,20,30]. Twins born from these pregnancies had the highest gestational age at birth and most neonatal outcomes were benign. Differences in perinatal outcomes were very marked when comparing type I vs. type II or III, whereas they were smaller when comparing type II and III.

Type II is generally known to have the poorest prognosis, with the highest risk of fetal demise and neonatal morbidity; indeed, in our cohort, type II sFGR had the lowest in utero survival rate of both twins and the highest perinatal mortality rate.

If we consider birthweight centiles according to INTERGROWTH-21st [31], the average weight of the smaller twin is below the 5th centile for gestational age in all three types; this confirms our diagnostic accuracy. As regards the bigger twin, in type I and II the average weight corresponds to about the 50th centile, while in type III the average weight reaches the 75th centile. There seems to be no obvious reason for this difference and no consideration on this matter is available in the literature.

Type III sFGR was associated with a marked neonatal morbidity, even worse than type II. Type III pregnancies delivered at a smaller gestational age, which most likely had a negative influence on adverse outcomes, as suggested by Curado [12]. Remarkably, about one third of type III sFGR newborns had malformations, most of which were ventricular septal defects, a much higher rate than type I and II. A three-fold higher prevalence of congenital heart defects in MCDA twin pregnancies complicated by sFGR was previously reported [32], though no significant difference in the three types was found.

In our series there were only two cases of fetal demise and one miscarriage, all occurring in the type II subgroup. Accordingly, type II sFGR twins resulted to have the lowest in utero survival rate and the highest risk of single fetal demise, a finding comparable to that reported by Rustico [14]; type I and type III sFGR had 100% in utero survival of both twins. The risk of fetal demise was significantly higher in type II compared with type I sFGR, while the difference between type II and type III did not result significant, coherent with what was reported by Buca [30]. Overall, our data suggest that type III sFGR has lower risk of fetal demise than what is classically believed, in agreement with Shinar [33]. We also confirm a significantly increased perinatal mortality in type II compared to type I and III, with a nearly three-fold and two-fold increase in the risk of perinatal death, respectively. Noticeably, there were no cases of fetal demise of both twins, unlike most other reports [9,14,27,30]. Batsry [28] also reported a 100% in utero survival rate of both twins in type I sFGR and found no significant difference in fetal demise rates between the three types. However, he did find a significant difference in overall perinatal mortality between the three types.

It must also be remarked that, in our series, cord occlusion was offered up to 23 weeks of gestational age, earlier than in most other studies (Gratacos < 28 weeks; Batsry < 26 weeks) [9,27]. This could have an impact on reported outcomes, with higher possibility of adverse events, including fetal demise. Despite this, our perinatal outcomes were comparable to those reported in the literature. This could be explained by the application of an extremely strict prenatal follow-up protocol involving serial US assessments and hospitalization of severe cases, similar to Batsry [28].

In most other studies, delivery of type II and III sFGR was scheduled at 31–32 weeks of gestational age. Of note, we scheduled delivery up to 34 weeks of gestational age in selected cases if no deterioration of DV Doppler or alteration in cardiotocography was detected, with no apparent increase in adverse outcomes. This agrees with Shinar [33], whose study reported a very low risk of fetal demise (0.7%) and a low risk of adverse neonatal outcome in type III sFGR pregnancies which continue until 34 weeks. However, it should be noted that our numbers are rather small and the study is underpowered to draw conclusions on the matter. Larger and prospective studies are required to further investigate this issue. A recent survey by the FERN Study Team [34] confirmed a significant and alarming heterogeneity in the management of sFGR in monochorionic twin pregnancies, especially in early-onset type II and III FGR cases. Thus, there is a strong need for clear and unambiguous guidelines on the matter in order to optimize and standardize clinical management of these complicated pregnancies.

Gestational age at diagnosis, which was claimed to be associated with adverse outcomes [12,28,29], was not associated with a poorer prognosis in the present study.

The main strength of the present work is to be a single-centre study comprising a relatively large cohort in which all pregnancies were followed-up by the same team, applying a unified management protocol. Limitations of the study include its retrospective nature and the 13-year time range. To this purpose, the team of sonographers did not change over this period, thus assuring a fairly homogeneous management. Another limitation of the study is the lack of statistical adjustment for potential confounders such as maternal age, BMI, parity and mode of conception. However, since the groups are not significantly different, there is no reason to assume this would be a crucial factor in influencing the results of the present study.

## 5. Conclusions

In conclusion, our study confirms that, though the classification of sFGR in three types according to UA flow pattern correlates with perinatal outcome, one third of cases show a shift in the flow pattern throughout pregnancy. Such a shift implies a need for close monitoring of UA Doppler, which can guide clinical management and timing of delivery: if Doppler monitoring is not scheduled correctly, important changes in Doppler flow pattern could be missed. Sudden deterioration of the UA and/or DV Dopplers can reliably guide clinicians on the delicate matter of timing of delivery.

Also, despite the limitation of a small sample, our data suggest that type III sFGR cases may have a lower risk of fetal demise than traditionally thought. This finding should be further explored in larger studies in order to be confirmed.

The main challenge remains finding the optimal balance between the risks of fetal demise and premature birth. Indeed, it might be worth it to investigate the option of reaching higher gestational ages. Further randomized study may gather data supporting the option to reach 34 weeks of GA even in type III sFGR, aiming to minimize adverse outcomes related to very premature birth, without increasing perinatal death rates.

## Figures and Tables

**Table 1:** Maternal characteristics of the 103 pregnant women included in the study, classified according to the type of sFGR. Data are shown as mean ± SD or as number and percentage (in parenthesis). BMI = Body Mass Index.

Maternal Characteristics	Type I	Type II	Type III	*p*-Value
Age (years)	32.3 ± 5.1	31.6 ± 5.4	32.4 ± 4.6	0.828
BMI (kg/m^2^)	21.6 ± 4.7	23.1 ± 3.8	23.1 ± 4.1	0.237
Parity > 1	20 (33.3%)	8 (30.8%)	7 (41.1%)	0.373
Spontaneous conception	52 (86.7%)	23 (88.5%)	17 (100%)	0.645
Caucasian ethnicity	53 (88.3%)	23 (88.5%)	16 (94.1%)	0.227

**Table 2:** Changes in UA Doppler flow pattern throughout gestation. Data are shown as number and percentage (in parenthesis). Re-classification: * 6 cases progressed to type III, 14 progressed to type II; + 6 cases progressed to type III, 6 cases changed to type I; § 3 cases changed to type I; and 1 case changed to type II.

	Type I	Type II	Type III
First consultation	71 (68.9%)	23 (22.4%)	9 (8.7%)
Re-classified	20 * (28.2%)	12 + (52.2%)	4 § (44.4%)
Last consultation	60 (58.3%)	26 (25.2%)	17 (16.5%)

**Table 3:** Obstetric outcomes in pregnancies with type I (*n* = 60), II (*n* = 26) and III (*n* = 17) sFGR. Data are shown as mean ± SD or as number and percentage (in parenthesis).

	Type I	Type II	Type III
Gestational age at diagnosis (weeks)	19.7 ± 4.9	17.8 ± 2.9	16.6 ± 1.3
Spontaneous improvement	19 (31.7%)	3 (11.5%)	2 (11.8%)
Delivery due to non-reassuring fetal monitoring	34 (56.7%)	19 (73.1%)	11 (64.7%)
Gestational age at birth (weeks)	34.4 ± 1.2	32.2 ± 2.1	31.9 ± 1.8
Preterm birth < 28 weeks	0	2 (7.7%)	0
Preterm birth < 32 weeks	3 (5.0%)	12 (46.2%)	14 (82.4%)
Urgent Cesarean delivery	10 (16.7%)	7 (26.9%)	7 (41.2%)
Single fetal demise	0	2 (7.7%)	0
In utero survival of both twins	60 (100%)	23 (88.5%)	17 (100%)

**Table 4:** Obstetric outcomes in pregnancies with type I (*n* = 60), II (*n* = 26) and III (*n* = 17) sFGR, comparison between subgroups. Data are shown as mean ± SD or as number and percentage (in parenthesis).

	Type I vs. II		Type II vs. III		Type I vs. III	
	*p*-Value	OR (95%CI)	*p*-Value	OR (95%CI)	*p*-Value	OR (95%CI)
Gestational age at diagnosis (weeks)	0.190	–	0.187	–	0.030	–
Spontaneous improvement	0.178	0.3 (0.1–1.5)	0.893	1.2 (0.1–9.4)	0.181	0.3 (0.1–1.8)
Delivery due to non-reassuring fetal monitoring	0.190	2.2 (0.7–6.8)	0.675	0.7 (0.1–3.5)	0.523	1.5 (0.4–5.7)
Gestational age at birth (weeks)	<0.001	–	0.571	–	<0.001	–
Preterm birth < 28 weeks	0.028	3.3 (1.4–5.7)	0.261	0.8 (0.1–2.5)	–	–
Preterm birth < 2 weeks	<0.001	5.6 (3.8–10.2)	0.117	2.9 (0.8–11.9)	<0.001	6.8 (5.5–20.3)
Urgent cesarean delivery	0.330	1.8 (0.5–5.8)	0.290	2.1 (0.5–8.1)	0.029	3.7 (1.2–12.6)
Single fetal demise	0.127	1.8 (0.7–3.1)	0.432	0.7 (0.3–2.3)	–	–
In utero survival of both twins	0.007	0.6 (0.2–0.8)	0.146	1.9 (0.7–3.4)	1	–

**Table 5:** Neonatal outcomes in cases with type I (*n* = 114), II (*n* = 43) and III (*n* = 34) sFGR. Data are shown as mean ± SD or as number and percentage (in parenthesis). RDS = respiratory distress syndrome; IVH = intraventricular hemorrage. NICU = Neonatal Intensive Care Unit. * Data are expressed as percentage of the total number of fetuses (120 type I, 52 type II, 34 type III).

	Type I	Type II	Type III
Fetal weight discordance (%)	18.5 ± 9.8	21.5 ± 12.8	24.2 ± 13.8
Birth weight (larger twin, g)	2043 ± 316	1595 ± 417	1765 ± 346
Birth weight (smaller twin, g)	1679 ± 313	1257 ± 384	1236 ± 299
NICU admission	76 (66.7%)	37 (86.0%)	34 (100%)
Length of NICU stay (days)	18.7 ± 15.8	23.4 ± 15.5	31.8 ± 29.0
RDS	51 (44.7%)	30 (69.8%)	29 (85.3%)
Apgar score 1′ < 5	11 (9.6%)	6 (14.0%)	8 (23.5%)
Apgar score 5′ < 7	10 (8.8%)	6 (14.0%)	8 (23.5%)
IVH (III-IV grade)	0	1 (2.3%)	3 (8.8%)
Sepsis (early onset)	1 (0.9%)	0	1 (2.9%)
Sepsis (late onset)	1 (0.9%)	1 (2.3%)	3 (8.8%)
Malformations	13 (11.4%)	3 (7.0%)	10 (29.4%)
Neonatal mortality	2 (1.8%)	1 (2.3%)	1 (2.9%)
Perinatal mortality *	2 (1.7%)	4 (7.7%)	1 (2.9%)

**Table 6:** Statistical comparison of neonatal outcomes in cases with type I (*n* = 114), II (*n* = 43) and III (*n* = 34) sFGR. RDS = respiratory distress syndrome; IVH = intraventricular hemorrhage. NICU = Neonatal Intensive Care Unit.

	Type I vs. II		Type II vs. III		Type I vs. III	
	***p*** **-Value**	**OR (95%CI)**	***p*** **-Value**	**OR (95%CI)**	***p*** **-Value**	**OR (95%CI)**
Fetal weight discordance (%)	0.253	–	0.517	–	0.046	–
Birth weight (larger twin, g)	<0.001	–	0.169	–	0.002	–
Birth weight (smaller twin, g)	<0.001	–	0.483	–	< 0.001	–
NICU admission	0.016	3.1 (1.2–7.9)	0.023	2.2 (1.2–6.7)	< 0.001	2.8 (1.3–4.2)
Length of NICU stay (days)	0.986	–	0.129	–	0.003	–
RDS	0.005	2.9 (1.3–6.0)	0.110	2.5 (0.8–7.9)	< 0.001	7.2 (2.6–19.8)
Apgar score 1’ <5	0.439	1.5 (0.5–4.4)	0.279	1.9 (0.6–6.1)	0.034	2.8 (1.1–7.8)
Apgar score 5’ <7	0.339	1.7 (0.6–4.9)	0.279	1.9 (0.6–6.1)	0.021	3.2 (1.2–8.9)
IVH	0.098	2.6 (0.4–4.8)	0.411	2.1 (0.3–13.6)	<0.001	4.2 (1.3–6.3)
Early onset sepsis	0.543	0.9 (0.1–8.9)	0.241	6.1 (0.6–24.3)	0.320	3.8 (0.3–16.9)
Late onset sepsis	0.459	2.8 (0.7–3.8)	0.361	2.4 (0.6–6.7)	0.048	7.8 (1.4–10.7)
Malformations	0.437	0.6 (0.2–2.2)	0.007	5.9 (1.5–18.8)	0.006	3.5 (1.4–9.1)
Neonatal death	0.816	1.3 (0.2–15.1)	0.423	2.6 (0.2–12.3)	0.193	3.5 (0.5–22.6)
Perinatal mortality	0.028	3.8 (1.4–4.6)	0.042	1.9 (1.2–2.4)	0.234	1.4 (0.9–3.8)

**Table 7:** Perinatal outcomes between early- (<24 weeks, *n* = 89) and late-onset (>24 weeks, *n* = 14) sFGR cases. TTTS = twin to twin transfusion syndrome.

	Early Onset	Late Onset	*p*-Value
Spontaneous improvement	24 (26.9%)	0	0.098
Superimposed TTTS	6 (6.7%)	2 (14.2%)	0.730
Delivery due to non-reassuring fetal monitoring	48 (53.9%)	14 (100%)	0.054
Gestational age at birth (weeks)	33.4 ± 2.1	33.3 ± 1.6	0.860
Urgent cesarean delivery	25 (28.1%)	3 (21.4%)	0.714
In utero survival of both twins	86 (96.6%)	100%	0.485
Fetal weight discordance (%)	18.8 ± 12.0	22.0 ± 11.3	0.463

## Data Availability

The data presented in this study are available upon request from the corresponding author. The data are not publicly available due to privacy reasons.

## Abbreviations

The following abbreviations are used in this manuscript:

sFGR	Selective fetal growth restriction
TTTS	Twin to twin transfusion syndrome
MCDA	Monochorionic diamniotic
NICU	Neonatal intensive care unit
GA	Gestational age
UA	Umbilical artery
MCA	Middle cerebral artery
RDS	Respiratory distress syndrome
